# Molecular dynamic simulation reveals the inhibiting impact of Rhein on wild-type and P29S-mutated Rac1

**DOI:** 10.3389/fmolb.2024.1414197

**Published:** 2024-08-05

**Authors:** Negar Etebar, Seyed Hootan Hamidi, Saghi Naderpour, Omar Abouali, Seyedeh Harir Hamidi, Behnam Hajipour-Verdom, Alireza Zali, Mozhgan Alipour, Milad Rahimzadegan

**Affiliations:** ^1^ Functional Neurosurgery Research Center, Shohada Tajrish Comprehensive Neurosurgical Center of Excellence, Shahid Beheshti University of Medical Sciences, Tehran, Iran; ^2^ Faculty of Pharmacy, Eastern Mediterranean University, Famagusta, Cyprus; ^3^ Acharya BM Reddy College of Pharmacy, Rajiv Gandhi University of Health Sciences, Bangalore, India; ^4^ Faculty of Pharmacy, Cyprus International University, Nicosia, Cyprus; ^5^ Al-Ameen College of Pharmacy, Rajiv Gandhi University of Health Sciences, Bangalore, India; ^6^ Integrative Oncology Department, Breast Cancer Research Center, Motamed Cancer Institute, Academic Center for Education, Culture and Research (ACECR), Tehran, Iran; ^7^ Department of Biophysics, Faculty of Biological Sciences, Tarbiat Modares University, Tehran, Iran

**Keywords:** cancer, Rac1, Rhein, P29S mutation, molecular dynamics simulation

## Abstract

Ras-related C3 botulinum toxin substrate 1 (Rac1) is a small GTPase belonging to the Rho family. It acts as a binary molecular switch regulating several cellular functions, including cell adhesion and migration. Malfunctions due to the P29S mutation in Rac1 increase the stability of the activated form of Rac1. This sustained activation can drive aberrant cellular processes associated with cancer, such as cell proliferation, survival, and migration. Therefore, finding an inhibitor that can inhibit the mutant form of the protein is very important. Rhein, a natural compound with diverse pharmacological properties, has been studied in relation to Rac1. However, specific interactions between Rhein and Rac1 have not been examined. In this study, we investigated the potential of Rhein, a natural compound, as an inhibitor of two forms of Rac1: the wild type and the P29S mutation, using molecular dynamics simulations. Results indicated that the P29S mutation led to structural changes in the Rac1 protein, which resulted in greater accessibility of the Rhein to the active site. In addition, the binding energy of Rhein to mutant Rac1 was more negative than the native protein. Therefore, it seems that the Rhein has a better inhibitory effect on the P29S-mutated form of the Rac1 protein.

## Introduction

Cancer is a complex disease and a particularly problematic condition because of its ability to evade growth suppressors, resist cell death, sustain proliferative signaling, metastasize, and invade. All of these are aspects that have been studied extensively (Hanahan and Weinberg, 2011; Hajipour et al., 2018; Weiss et al., 2022). Ras-related C3 botulinum toxin substrate 1 (Rac1) is a promising emerging target due to its critical role in cancer progression, as its dysregulation has been reported to be implicated in various forms of cancer (Liang et al., 2021; Ma et al., 2023).

Rac1 is a small GTPase belonging to the Rho family. It acts as a binary molecular switch. It is present in two forms: an active GTP-bound form (on) and an inactive GDP-bound form (off) (Tebar et al., 2018; Lin et al., 2023). This switch function is regulated by guanine nucleotide exchange factors (GEFs) as the activators and GTPase activating proteins (GAPs) as the inactivators. Rac1 plays a critical role in the regulation of multiple cellular processes. Its activity as a modulator of the cytoskeleton affects phagocytosis, axonal growth, adhesion, migration, cell differentiation, cell growth, cell cycle regulation pathways, and cell–cell adhesion (Jaffe and Hall, 2005; Rossman et al., 2005; Margiotta and Bucci, 2019).

Structurally, Rac1 is a monomeric protein that contains 189 amino acids and weighs an estimated 21 kDa. It switches between an inactive GDP-bound state and an active GTP-bound state. It has three domains: the N-terminal, central, and C-terminal. The N-terminal domain includes amino acids 1–19 and plays a critical role in Rac1 localization and trafficking, as well as interaction with other proteins (Kumar et al., 2013; Ueyama et al., 2013; Rani et al., 2023).

**FIGURE 1:**
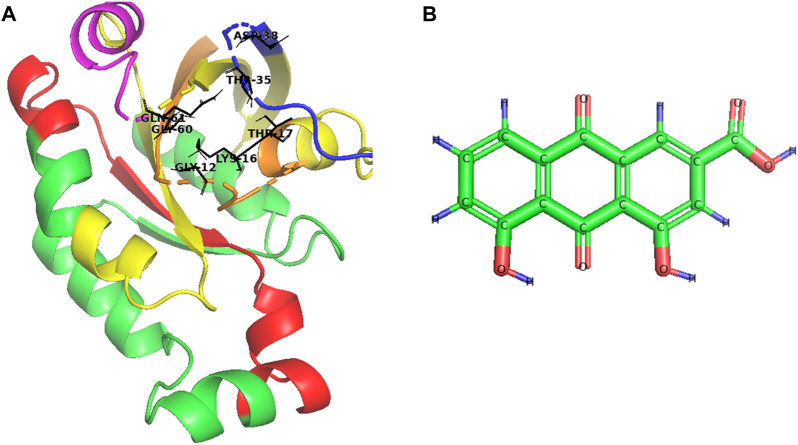
Schematic structure of **(A)** Rac1 and **(B)** Rhein (C_15_H_8_O_6_). In Rac1, the GTPase domain is yellow, the switch I region is blue, the switch II region is pink, the effector-binding region is red, the N-terminal is orange, and the binding pocket is black.

The central domain (amino acids 20–126) has four subdomains: the GTPase domain (amino acids 20–91), the switch I region (amino acids 29–44), the switch II region (amino acids 56–75), and the effector-binding region (amino acids 100–126). The GTPase domain is the “Achilles heel” of Rac1 activity, controlling the binding and hydrolysis of GTP. In addition, the critical amino acids in the binding pocket of Rac1 include Gly12, Lys16, Thr17, Thr35, Asp38, Gly60, and Gln61 (Figure 1A). The switch I and II regions change conformation in response to GTP binding or hydrolysis, which can activate or deactivate Rac1 for downstream signaling. The effector-binding region induces downstream effector proteins when Rac1 is bound to GTP, which in turn initiates various Rac1-dependent signaling pathways (Matos et al., 2000; Grizot et al., 2001; Jezyk et al., 2006; Koehn et al., 2023).

The C-terminal domain of Rac1 consists of nine amino acids and is involved in interactions with GEFs and GAPs. This domain is critical in regulating its activity and interactions with other proteins, as well as Rac1 membrane localization. Ions can also optimize Rac1’s function as co-factors. Magnesium ions can facilitate GTP binding in Rac1, while calcium ions can optimize Rac1 activity by interacting with downstream effector molecules (Menard and Snyderman, 1993; Van Hennik et al., 2003; Lam and Hordijk, 2013).

Activation of Rac1 changes undergo mutations. One of the important mutations is the P29S substitution. This mutation occurs in the switch I region and causes a bending of the backbone of the protein (a kink) at the proline residue located at position 29. The kink increases the stability of Rac1-GTP, thereby amplifying Rac1 signaling. This aggressive mutation has been implicated in several cancers, such as melanoma, bladder cancer, and head and neck squamous cell carcinoma (Worthylake et al., 2000; Watson et al., 2014; Rajendran et al., 2016). Rac-1 and its mutated derivatives play a significant role in the progression of various cancers through their molecular signaling (Bid et al., 2013). As a result, investigating Rac-1 inhibitors can be beneficial in finding potential anticancer drugs.

Natural agents and their analogs can be candidates for Rac-1 antagonist drug discovery with various therapeutic activities due to the phytochemicals they contain. Rhein (4,5-dihydroxyanthraquinone-2-carboxylic acid) is a lipophilic anthraquinone phytochemical known as rhubarb yellow with the structure shown in Figure 1B. It has a molecular formula of C_15_H_8_O_6_ (Li et al., 2020; Xiong et al., 2023).

Rhein has various therapeutic potentials, including anti-inflammatory, antioxidant, antimicrobial, anticancer, hepatoprotective, nephroprotective, cardiovascular, and neuroprotective potential. These effects may be mediated through the regulation of various signaling pathways, such as inhibiting Rac-1 and interrupting downstream signaling. However, more research is needed to fully understand the mechanism and clinical applications of Rhein in cancer treatment (Henamayee et al., 2020; Cheng et al., 2021).

Resent study showed that Rhein binds to Rac-1 switch regions on several amino acids, such as Tyr64, Ser129, Lys147, Asp150, Asn151, Ser153, Lys155, Thr156, and Glu183, leading to Rac-1 conformational changes (Li et al., 2020). This binding suppresses Rac1 activity. Moreover, Rhein has been found to inhibit the signaling pathways that Rac-1 activates, including the Jun N-terminal kinase (JNK) and p38 mitogen-activated protein kinase pathways. Various cellular processes are affected through this inhibition, such as cell proliferation, migration, and survival (Zhen et al., 2011; Hao et al., 2019; Zhao et al., 2023). In addition, Rhein also inhibits aggressive mutated forms of Rac-1, such as the P29S mutation (Su et al., 2019; Liang et al., 2021).

These properties introduced Rhein as a potent anticancer agent; however, the interaction of this Rhein with Rac1 needs to be analyzed. We used molecular dynamics (MD) simulation to examine the potency of Rhein for inhibiting two forms of Rac1: the wild type and the P29S mutation. Our results indicated that Rhein binds to the P29S-mutated form of Rac1 with higher affinity than the wild-type form. Furthermore, the root mean square deviation (RMSD) analysis indicated that the binding of the Rhein molecule to the P29S-mutated Rac1 leads to more significant structural changes in this protein. Generally, it seems that the inhibitory effect of Rhein on the P29S-mutated form of Rac1 protein is greater than its effect on the wild-type form. These results highlight the potential of Rhein as a promising therapeutic agent for targeting the P29S-mutated Rac1 in anticancer treatments.

## Materials and methods

### Molecular docking

Three-dimensional (3D) structures of native and P29S-mutated Rac1 were obtained from the Protein Data Bank with PDB ID: 3TH5 and 3SBD, respectively. The 3D structure of Rhein was obtained from the PubChem database (https://pubchem.ncbi.nlm.nih.gov/). AutoDockTools 1.4.6 was used to dock Rhein to native and P29S-mutated Rac1 using the Lamarckian genetic algorithm. The Rhein being docked was also kept flexible.

### Molecular dynamic simulation

All of the atom MD simulations were performed using the GROMACS 4.5.4 package (Van Der Spoel et al., 2005) for the native Rac1 form, the native Rac1 form with Rhein (NR), and the P29S-mutated Rac1 form with Rhein (MR) complexes during a 100-ns simulation, and each simulation was repeated three times (n = 3). These systems were solvated in a rectangular box with TIP3P water, and a physiological concentration of Na^+^/Cl^−^ (0.15 M) was added to neutralize the systems. The protonation state of proteins was pH = 7. Then, minimization was done by the steepest descent algorithm with a maximum force <1000.0 kJ/mol/nm. The V-rescale thermostat and Parrinello–Rahman methods were employed to couple temperature (310 K) and pressure (1 bar) within NVT and NPT ensembles, respectively. Finally, the systems were run with periodic boundary conditions and a time step of 2 fs implementing a CHARMM36 force field (MacKerell et al., 1998) during 100-ns and 200-ns simulations. The particle mesh Ewald method was used to compute electrostatic interactions (Alipour et al., 2022; Khodayari et al., 2022).

### Calculations of binding free energy and dissociation constant

The degree of the interaction strength between two molecules, such as a ligand and a receptor or a substrate and an enzyme, is expressed in terms of the dissociation constant (Kd). There is a relationship between Kd and binding free energy (ΔG). The relationship is inverse and logarithmic, according to Eq. 1.

∆G=−RT⁡ln⁡Kd, (1)

where ΔG is the binding free energy, R is the gas constant, and T is the temperature in Kelvin. Moreover, ΔG was calculated through molecular mechanics Poisson–Boltzmann surface area method (MM-PBSA). A lower Kd value indicates a stronger interaction, meaning that the molecules have a higher affinity for each other. In contrast, a more negative ΔG shows a more stable complex, as the system is energetically favorable in the bound state (Alipour et al., 2022). Therefore, Kd and ΔG were calculated between the Rhein and the Rac1 protein in the NR and MR complexes during 100-ns simulations.

## Results and discussion

### Binding model of Rhein to Rac1

**FIGURE 2:**
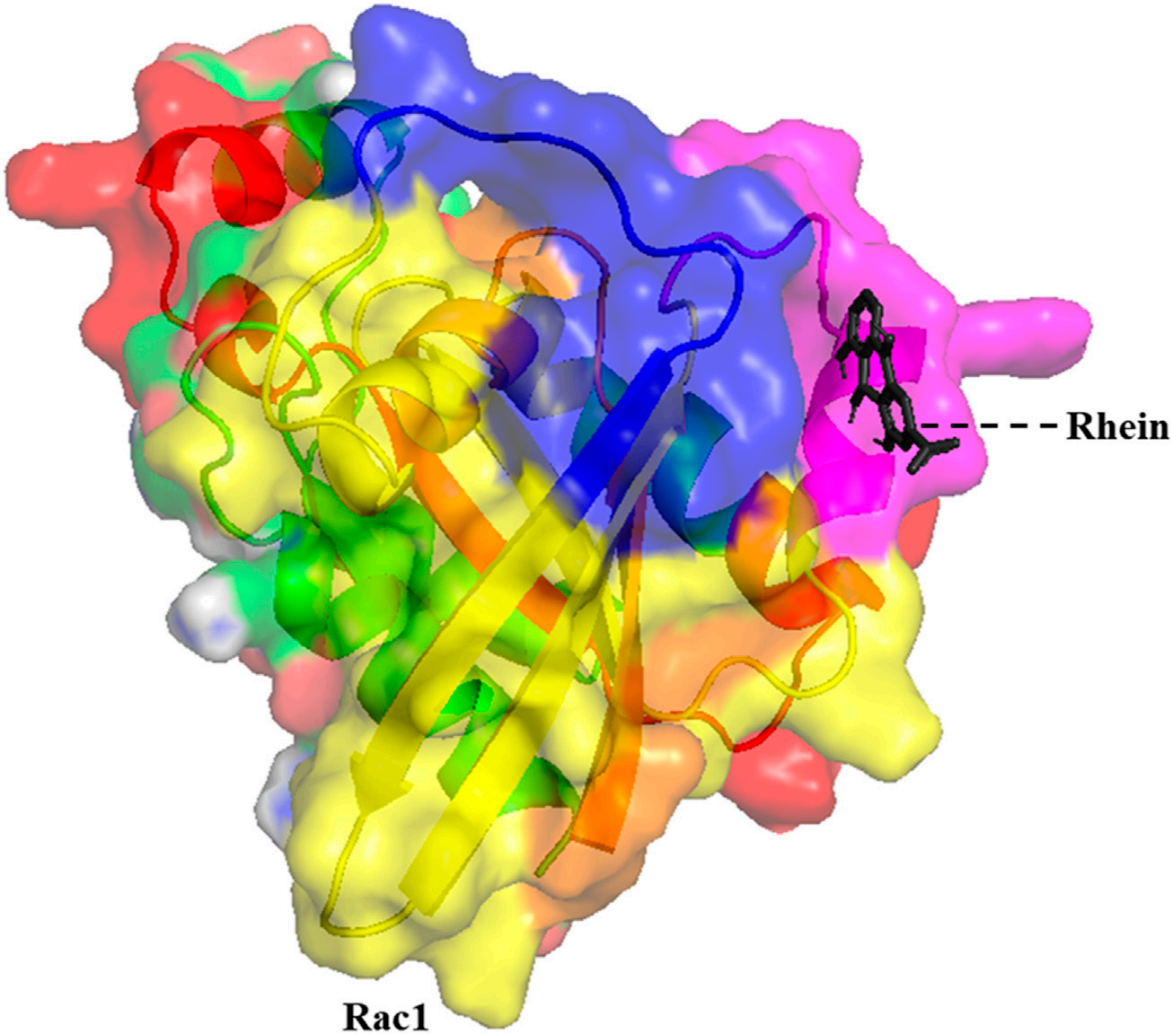
The 3D structure of the minimum energy conformation of Rhein docked with Rac1 (The GTPase domain is yellow, the switch I region is blue, the switch II region is pink, the effector-binding region is red, and the N-terminal is orange).

In this study, we examined the inhibitory role of Rhein in the wild type and cancer-associated P29S mutation in the Rac1 protein. The interaction between Rhein and specific residues in the Rac1 binding pocket (including Gly1, Lys16, Thr17, Thr35, Asp38, Gly60, and Gln61) is crucial for its inhibitory function (Koehn et al., 2023). The molecular docking analysis showed that Rhein binds with higher affinity to the mutated protein than to the wild-type protein. In the wild-type Rac1 protein, binding energy was ∼6.64 kcal/mol, whereas in the mutant, it was found to be ∼4.85 kcal/mol. As shown in Figure 2, in the wild-type form of Rac1, Rhein is located between the Switch 1 and Switch 2 regions and interacts with the amino acids of this region, which include Thr35, Val36, Asp38, Gly60, Gln61, Glu62, Asp65, Ala97, Leu100, Ile117, Lue127, and Phe141. In the mutated form, this ligand was also located in the same region with a slight rotation compared to the wild-type form. Protein and ligand interactions frequently trigger notable alterations in their structure. MD simulations demonstrated the molecular shifts linked to the affinity of the wild-type and mutant proteins for ligand interaction.

### Stability and flexibility

RMSD is a crucial metric used in MD simulations to quantify the structural differences between a reference structure (typically an initial or target structure) and subsequent structures generated during the simulation. RMSD provides insights into how much a molecular structure fluctuates or deviates from its initial conformation during the simulation. It is a useful measure to assess the stability and reliability of simulations, as well as to compare different simulation trajectories or to evaluate the binding affinities of ligands to proteins. Lower RMSD values indicate greater structural similarity between frames, suggesting more stable or consistent simulations, while higher RMSD values suggest greater structural variation or instability (Kufareva et al., 2012).

**FIGURE 3:**
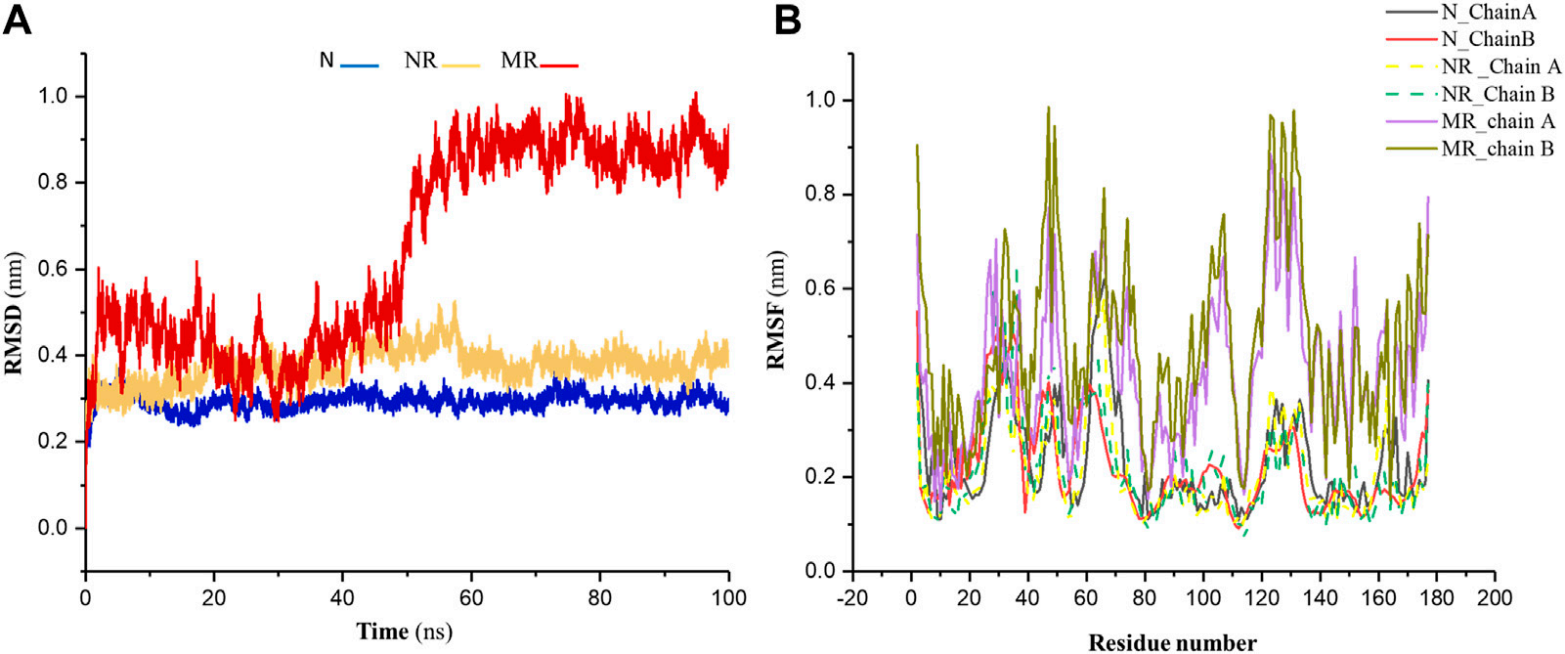
**(A)** The RMSD of backbone for the native Rac1 form (N), the native Rac1 form with Rhein (NR), and the P29S-mutated Rac1 form with Rhein (MR) complexes. **(B)** The RMSF profile for chains A and B in the N, NR, and MR complexes during 100-ns simulations.

Backbone-RMSD was calculated for the native Rac1 form (N), the native Rac1 form with Rhein (NR), and the P29S-mutated Rac1 form with Rhein (MR) complexes during 100-ns simulations. As shown in Figure 3A the results indicated that the value of RMSD in the MR complex increased more than in the other structures during the simulation. These values in the N, NR, and MR complexes were about 0.39 nm, 0.41 nm, and 0.91 nm, respectively. Indeed, the N and NR complexes were found to be more stable than MR, and their stability was further analyzed through root mean square fluctuation (RMSF) analysis. The results of the RMSF analysis showed a significant increase in the flexibility of the MR compared to the N and NR complexes (Figure 3B).

A simulation of the mutated protein was done for 200 ns to determine whether this change in stability of the MR complex is due to ligand binding to the protein or is a result of the mutation. As shown in Supplementary Figure S1, the result indicated that the RMSD of the mutant protein changed after about 50 ns, similar to the MR complex in that its value was about 0.9 nm. Therefore, it was determined that the alteration in the RMSD of MR compared to N and NR was not caused by the binding of ligands.

### Structural characteristics of protein

The solvent-accessible surface area (SASA) and the radius of gyration (Rg) are measures used in molecular biophysics to characterize the structure of molecules, particularly proteins. SASA refers to the area of a molecule’s surface that is accessible to solvent molecules. It is a measure of how much surface area of a molecule is available for interaction with other molecules in its surrounding environment, typically water molecules. A higher SASA value indicates that more of the molecule’s surface is exposed and available for interaction with solvent molecules, while a lower SASA value suggests that less surface area is accessible, possibly due to the molecule being more compact or buried within a protein structure. In contrast, Rg is a parameter of the compactness or spread of a molecule’s mass distribution around its center of mass. A larger radius of gyration suggests that the molecule is more extended, whereas a smaller radius indicates that the molecule is more compact (Durham et al., 2009; Kushwaha et al., 2021).

**FIGURE 4:**
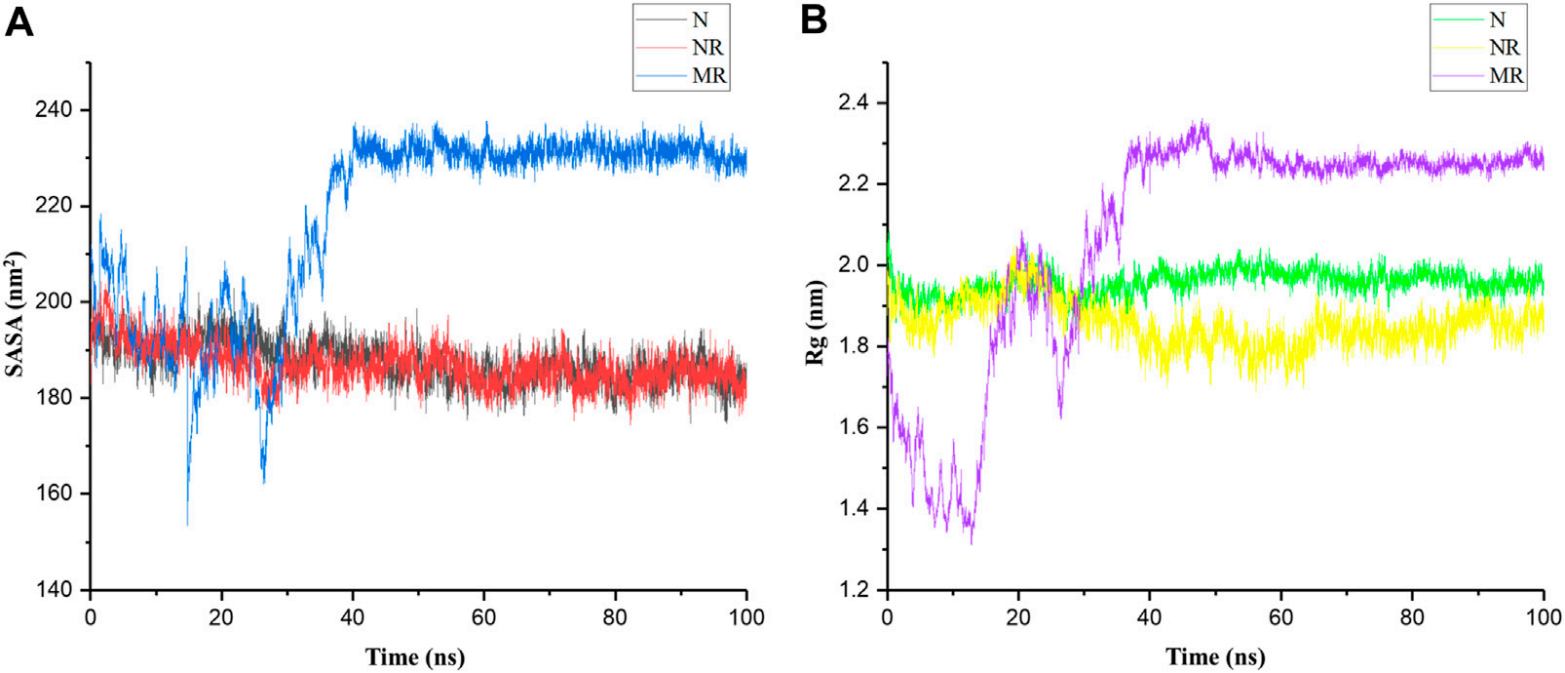
**(A)** Solvent-accessible surface area (SASA) and **(B)** radius of gyration (Rg) of the native Rac1 form (N), the native Rac1 form with Rhein (NR), and the P29S-mutated Rac1 form with Rhein (MR) complexes during 100-ns simulations.

Although SASA and Rg are related, in that they both provide information about the structure of a molecule, they capture different aspects of that structure. For example, a protein with a large SASA may have a more extended structure, leading to a larger Rg. However, it is also possible for a protein to have a large SASA but a relatively small Rg if the exposed surface area is distributed in a compact manner (Bagewadi et al., 2023). Therefore, for a better description of structural changes in the protein, both parameters were calculated for the N, NR, and MR complexes during 100-ns simulations. Results indicated that the SASA and Rg in the MR complex increased compared to the N and NR complex: the values of SASA and Rg were approximately 200 nm^2^ and 2 nm in the N and NR complexes and 230 nm^2^ and 2.23 nm in the MR complex, respectively (Figure 4). In other words, the increased SASA in the MR complex is accompanied by a decrease in the compactness of the structure.

The P29S mutation can significantly impact the structure and dynamics of Rac1, influencing its interactions with other molecules, including solvents. Replacing proline with serine may expose new regions of the protein surface to solvent molecules due to the differing size and chemical properties of these amino acids. Serine is larger and more polar than proline, potentially leading to changes in protein conformation or increased flexibility in certain regions of Rac1 (Figure 3B). Indeed, proline typically induces rigidity and kinks in protein structures because of its cyclic structure, so substituting it with serine could result in a more flexible region, thereby altering the local or overall conformation of Rac1. Therefore, it seems that a greater surface area of the P29S-mutated Rac1 is available for ligand binding. To investigate this result, the dynamic of Rhein inside the native and mutant proteins was examined by distance analysis during 100-ns simulations.

**FIGURE 5:**
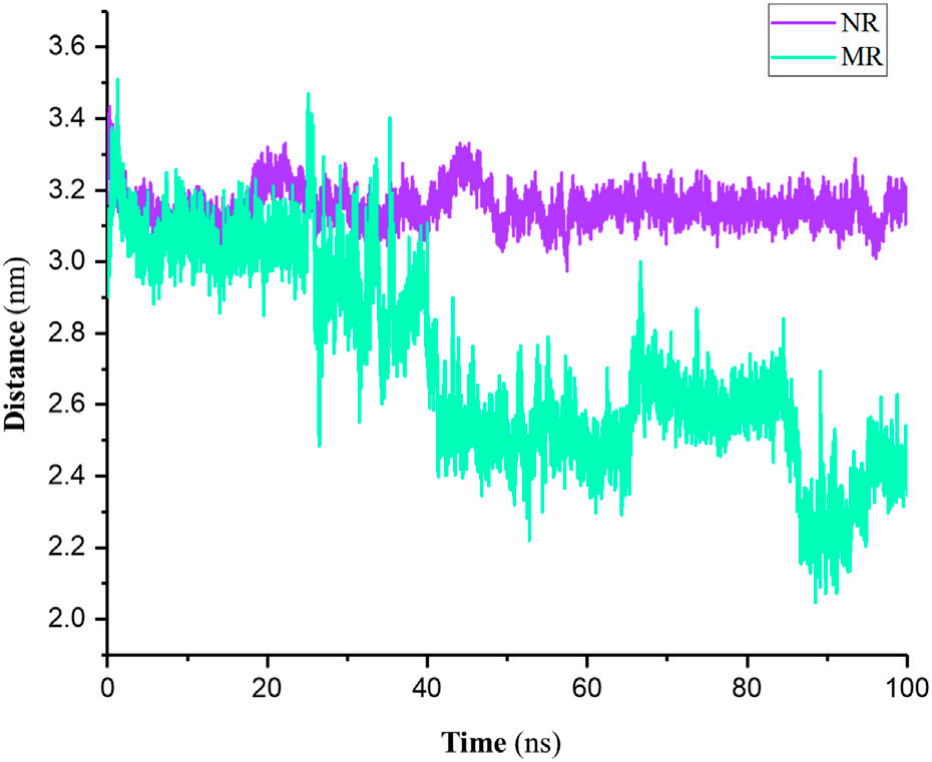
Distance of the Rhein relative to the COM of the active site in native Rac1 (NR) and P29S-mutated Rac1 form (MR) complexes during 100-ns simulations.

Distance analysis typically refers to the average distance traveled by atoms or molecules during a simulation relative to a reference (here, the center of mass (COM) of the active site). As shown in Figure 5 results indicated that in the NR complex, the distance of the Rhein relative to the active site of the protein remained constant. In other words, the position of Rhein did not change from its initial location in the active site. In the MR complex, however, the position of Rhein relative to the active site changed and interacted with more amino acids than the NR complex.

### Analysis of interaction

The interaction between a ligand and a protein is a crucial aspect of molecular recognition and binding that plays a fundamental role in various biological processes, such as enzyme catalysis, signal transduction, and drug action. The nature and strength of these interactions dictate the specificity and affinity of ligand binding to the protein. Some common interactions between a ligand and a protein include hydrogen bonds, van der Waals interactions, electrostatic interactions, and hydrophobic interactions. The specific combination of these interactions determines the overall binding affinity and specificity of the ligand–protein interaction (Alipour et al., 2022). The number of contacts (NC), hydrogen bonds (NHB), van der Waals forces, and electrostatic interactions were computed for the NR and MR complexes during 100-ns simulations.

**FIGURE 6:**
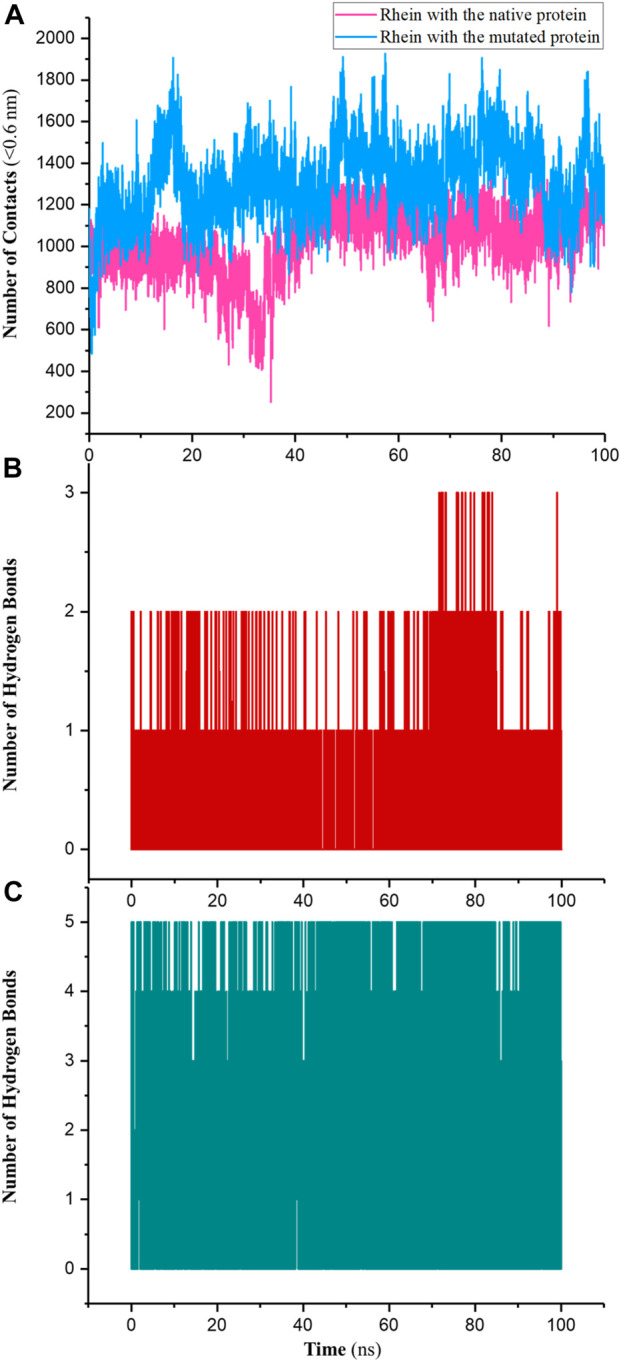
**(A)** Number of contacts (NC) between Rhein with the native (NR) and mutated Rac1 (MR) and number of hydrogen bonds (NHB) in **(B)** NR and **(C)** MR during 100-ns simulations.

NC typically refers to the number of pairwise interactions between atoms or molecules within a specified distance cutoff (in this work, 0.6 nm). These interactions could be covalent bonds, non-covalent bond interactions, or any other form of interaction. As shown in Figure 6A the results indicated that the NC between and P29S-mutated Rac1 was higher than the NC between Rhein with the native protein; the averages were approximately 1,450 and 1,210 in the MR and NR complexes, respectively. Like NC, the NHB in the MR complex was greater than the NHB in the NR complex: the maximum NHB between Rhein and P29S-mutated Rac1 was 5, while it was 3 in the native during 100-ns simulations (Figures 6B, C).

**FIGURE 7:**
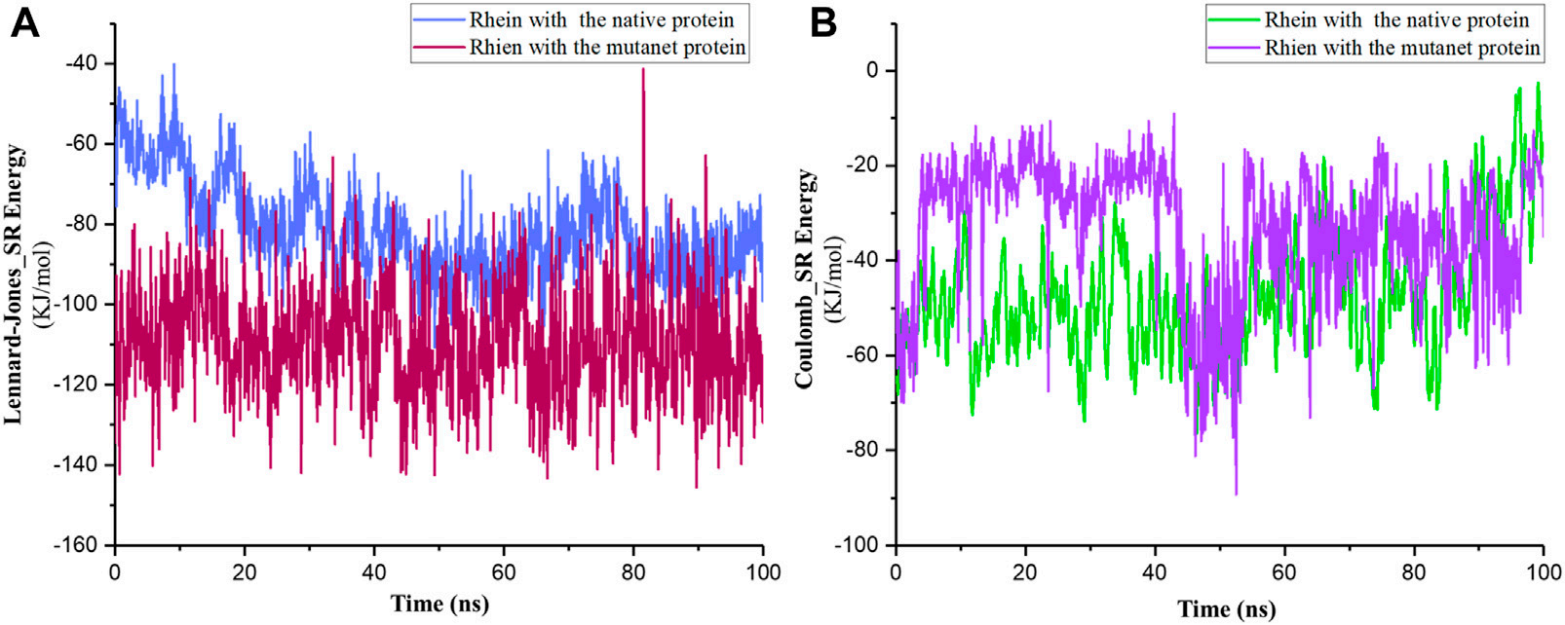
**(A)** Short-range Lennard–Jones and **(B)** Coulomb energies between Rhein with the native (NR) and mutated Rac1 (MR) during 100-ns simulations.

Additionally, results of van der Waals and electrostatic interactions showed that total energies between Rhein and P29S-mutated Rac1 were more negative than the total between Rhein and the native protein. The sums of the average energies in the MR and NR complexes were approximately −141.86 KJ/mol and −99.43 KJ/mol during 100-ns simulations (Figure 7). According to these results, it seems that the ligand is more tightly bound to P29S-mutated Rac1 than to the native Rac1.

### Binding energy analysis

In ligand–protein interactions, binding energy refers to the energy change associated with the formation of the complex between the ligand and the protein. This energy change results from various intermolecular forces such as hydrogen bonding, van der Waals interactions, electrostatic interactions, and hydrophobic interactions.

The binding energy quantifies the strength of interaction between the ligand and the protein. A more negative binding energy indicates a stronger binding affinity between the ligand and the protein, implying a more stable complex formation. Conversely, a less negative (or positive) binding energy suggests weaker binding and a less stable complex (Du et al., 2016). Eq. 1 shows an inverse relationship between ΔG and Kd; as ΔG becomes more negative (favorable), indicating stronger binding, Kd becomes smaller (approaches 0), meaning the dissociation constant decreases. ΔG and Kd were computed between Rhein and Rac1 in NR and MR complexes during 100-ns simulations.

The results showed that ΔG between the Rhein and Rac1 in the MR complex was smaller than in the NR complex. The values of this energy in the NR and MR complexes were −8.56 Kcal/mol and −12.33 Kcal/mol, respectively. On the other hand, the Kd values in the NR and MR complexes were 3.61 × 10^−2^ M and 8.39 × 10^−3^ M, respectively. Therefore, results indicated that Rhein was bound more tightly to the P29S-mutated Rac1 protein than the native Rac1.

## Conclusion

Rac1 is a small GTPase protein belonging to the Rho family of GTPases. It plays a crucial role in cellular signaling pathways that regulate diverse cellular processes. Mutations in the Rac1 gene have been associated with various types of cancers (Parri and Chiarugi, 2010). The P29S mutation in the Rac1 gene is a specific alteration that has been identified in various cancers and is associated with oncogenic activity. Recent studies show that mutations of the K-Ras protein lead to changes in its switch transformations and free energy profiles of the GDP- and GTP-bound K-Ras. Therefore, it is very important to examine the effects of this mutation on the structural and dynamic changes of the Rac1 protein (Chen et al., 2021; Chen et al., 2022). The P29S mutation leads to the constitutive activation of the Rac1 protein. Normally, Rac1 cycles between an inactive GDP-bound state and an active GTP-bound state in response to extracellular signals. However, the P29S mutation disrupts this cycling and locks Rac1 in its active state, even in the absence of activating signals (Davis et al., 2013; Acuner et al., 2021). Therefore, finding an inhibitor that can inhibit this form of the protein is very important. In this study, the inhibitory effect of Rhein on both native and P29S-mutated forms of the Rac1 protein was investigated.

The results showed that the P29S mutation leads to structural changes in the Rac1 protein. These structural changes include an increase in the protein’s accessible surface area and a decrease in structural compactness compared to native protein. These factors combine to result in greater accessibility of the Rhein molecule to the protein’s active site. Furthermore, the results demonstrated that the Rhein molecule binds to the P29S-mutated protein with a more negative energy than the native protein. Therefore, it seems that the Rhein molecule binds more strongly to the mutated Rac1 protein, leading to a more effective inhibition of this protein.

Rhein contains multiple hydroxy (^−^OH) groups in its structure, which can form hydrogen bonds with specific amino acid residues on the Rac1 protein, facilitating its binding to the protein’s active site. Additionally, Rhein possesses an aromatic ring system, allowing for pi–pi stacking interactions with aromatic amino acid residues on the protein, thereby enhancing the stability of the inhibitor–protein complex. Furthermore, Rhein exhibits a hydrophobic structure, enabling its hydrophobic moiety to interact with nonpolar regions of the Rac1 protein, thereby contributing to its overall binding affinity.

The presence of flexible bonds in the Rhein molecule permits it to adopt various conformations, potentially facilitating its accommodation within the binding pockets of the Rac1 protein. Additionally, the overall size and shape of Rhein, along with its structural resemblance to GTP, are crucial factors that enable it to fit into the binding site of the Rac1 protein, facilitating complementary interactions. In a recent study, the anticancer effects of modified Rhein structures were investigated. This study revealed that altering the functional groups of the Rhein molecule can modify its inhibitory potency, with an increase in the aromatic ring leading to enhanced inhibitory activity (Li et al., 2020). Based on these chemical features, Rhein may effectively bind to and inhibit the activity of Rac1, thereby modulating biological processes and potentially serving as a therapeutic agent in cancer diseases.

## Data Availability

The original contributions presented in the study are included in the article/Supplementary Material, further inquiries can be directed to the corresponding authors.
