# Supplementary figures and images for: Molecular dynamic simulation reveals the inhibiting impact of Rhein on wild-type and P29S-mutated Rac1
Source: Front Mol Biosci. 2024 Aug 5;11:1414197. doi: 10.3389/fmolb.2024.1414197 (PMC11330767; doi:10.3389/fmolb.2024.1414197)

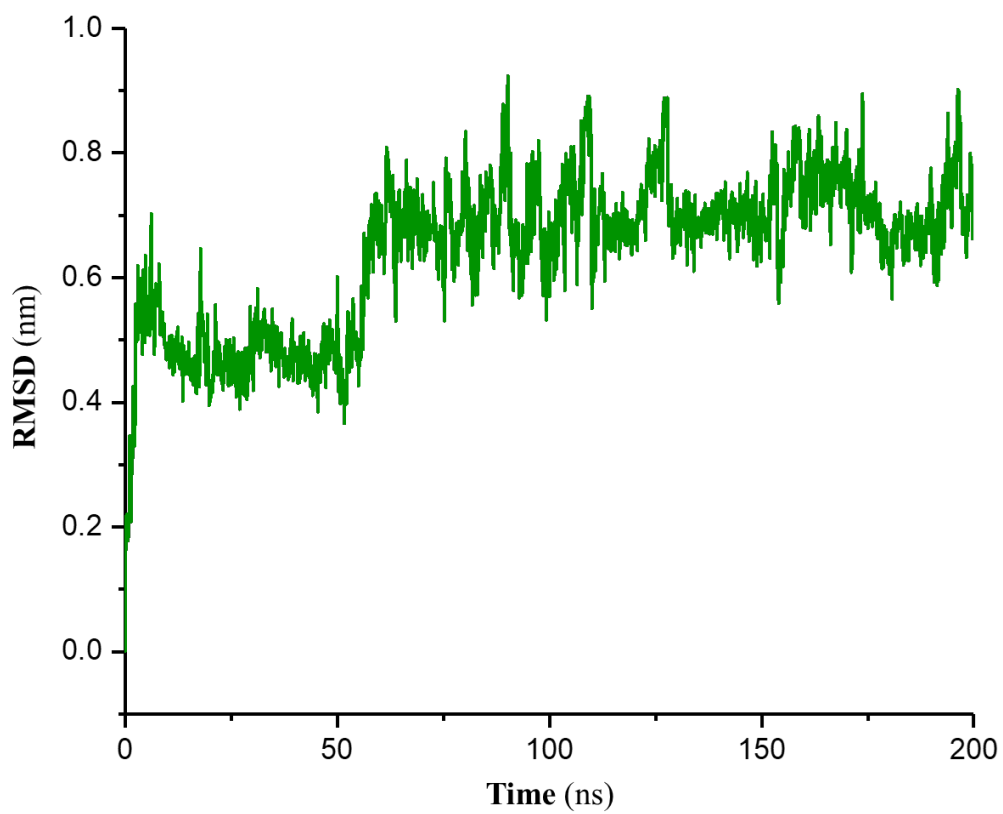

**Supplementary Figure S1.** RMSD of backbone for the P29S mutated Rac1 form during 200 ns simulation.

Supplement: Supplementary file 1 [file Image1.pdf]
